# The Veterinarian as a Sanitary Officer1Read before the Michigan Veterinary Medical Association, February, 1901.

**Published:** 1901-09

**Authors:** J. Drury

**Affiliations:** Ypsilanti, Mich.


### THE VETERINARIAN AS A SANITARY OFFICER.[1]

By J. Drury, V.S.,
YPSILANTI, MICH.

Boards of health for the protection of the human family are established institutions in this and, perhaps, in every other civilized country, but only in some localities are veterinarians in any way officially connected with them, and, we believe, to the inefficiency of said boards. A board of health, to thoroughly perform the work for which it is created, should include among its members a veterinarian—a veterinarian not simply because of his knowledge of certain diseases of animals which are known to be communicable to man and dangerous to health, but also by reason of his knowledge gained in the discharge of his everyday duties of certain unsanitary conditions which, if not dangerous to human life, can scarcely be considered favorable to perfect health either in man or in animals. The last report of the Food Commissioner for this State says: "We have a system of dairy inspection which is the most thorough and competent of any in the Union." If that is the case my sympathies are with the remaining States. A dairy inspector's report, published a short time ago, read something like the following: "Cows partly clean and partly dirty; stables partly clean and partly dirty." Now, as nothing was done to cause the cows to be kept cleaner, nor the stables less dirty, where was the good of the inspection? The employment of a resident inspector is the only practicable method for successfully remedying unsanitary conditions which exist in every section of the country

[1] Read before the Michigan Veterinary Medical Association, February, 1901.

in connection with our milk and meat supply. An outsider going through a modern butter or cheese factory, where everything is usually kept in as sanitary a condition as possible, or into the public section of a retail meat market, can have but a vague idea of the unwholesome conditions through which the respective foods have passed before reaching their apparently palatable state. Such is not the case, however, with the veterinarian, whose work brings him in contact with very different conditions in the primary handling of the foods mentioned.

In most large cities, it is true, a public inspector of milk and meat is employed, one where perhaps there should be a dozen, but what I wish to emphasize is the equal need of inspection in our towns, villages, and country at large, not only of milk and meat, but of the conditions under which they are furnished. Oleo or anti-oleomargarine bills are introduced into our State Legislatures for purposes best known to their promoters, but it is not an uncommon thing to hear a person remark, "I would much rather have oleomargarine on my table than butter from some dairies I know of," which can have only one interpretation, that the sanitary conditions are below the standard of the oleo factory. I believe less care is taken toward cleanliness by the milk handler since the introduction of the butter factory than under the old method of individual butter-making. It is now a question of butter fat, the quality of the butter being left entirely to the factory butter maker. Milk-inspection and meat-inspection is a most desirable object, both for town and country, but the inspection of animals used for food and for the production of milk, with their surroundings, is perhaps equally important, and the veterinarian is or should be the most competent person from the nature of his work or the establishing and controlling of a system of sanitary police. Whether the average veterinarian would care for the office or not is another story.

Next in importance to the inspection of dairies and dairy cows, meat and meat-furnishing animals, without doubt is the inspection of slaughter-houses. The public slaughter-house should not only be talked of, but should be here in reality, wherein all animals, at least all large domestic animals, should be killed, the same to be under competent inspection, which would insure not only sanitation but a practically perfect meat supply. I not only mean that animals slaughtered would be healthy or would otherwise be condemned and excluded from commerce, but that all animals would be endowed with a certain amount of flesh or be classed with the condemned list. As it is, animals are very often sold and shambled when they should be fed and stabled, but until the day of the public slaughter-house a remedy for existing conditions may be very hard to find. The question of the disposal of dead animals is a matter, too, that needs attention, more especially in the country. It should be compulsory that a person upon whose premises an animal dies should have the carcass either cremated or buried within a reasonable time. What too frequently happens, it is either fed to pigs or fowls, or is taken to the wood-lot to save funeral expenses.

The controlling of bovine tuberculosis is now a somewhat serious and difficult problem by reason of its prevalence and many-sided interests, both sanitary and financial, for which no one should be more fitted than the veterinarian from a pathological as well as a sanitary point of view. To kill and destroy the flesh of all animals merely because they respond to the tuberculin test seems rather too heroic for present needs, and, put into general practice, would mean an immense financial loss to the cattle owner. Dairy cows respond to the test sometimes whose milk is perfectly wholesome. Beef cattle, too, though showing tuberculosis per test, may to a greater or lesser extent be edible. If stock owners were assured that only animals totally unfit for food or for the production of food products would be destroyed they would more readily submit their animals to the tuberculin test; in fact, would be more or less in favor of it instead, as at present, avoiding it by every possible means—a possible argument in favor of the public slaughter-house.

Finally, competent supervision is required of all premises where meat or milk is produced, handled, or offered for sale, including dairies, butter and cheese factories, slaughter-houses and meat markets, together with the animals themselves and their immediate surroundings. What might or should be done by way of legislation to overcome some of the problems mentioned, together with various others which have not been, whether remedial laws should be municipal, State, or both, I leave with you, gentlemen, to suggest.

---

*Department of Canine, Feline, and Avian Surgery.*—Owing to the temporary absence of Dr. Cecil French in Europe and delay in the mails, this Department is unavoidably omitted in this number of the JOURNAL.

